# Clinical staff reported knowledge on the existence of clinical governance protocols or tools utilised in selected South African hospitals

**DOI:** 10.1371/journal.pone.0312340

**Published:** 2024-11-21

**Authors:** Nomfuneko Sithole, Wezile W. Chitha, Onke R. Mnyaka, Akhona B. A. Ncinitwa, Sibusiso C. Nomatshila, Xolelwa Ntlongweni, Kedibone Maake, Bongiwe E. Mkabela, Ntiyiso V. Khosa, Ziyanda B. Ngcobo, Nombulelo Chitha, Khanyisile Masuku, Sikhumbuzo A. Mabunda

**Affiliations:** 1 Department of Public Health, Walter Sisulu University, Mthatha, South Africa; 2 Mpumalanga Department of Health, Rob Ferreira Hospital, Mbombela, South Africa; 3 School of Population Health, University of New South Wales, Sydney, Australia; 4 George Institute for Global Health, University of New South Wales, Sydney, Australia; University of Pretoria, SOUTH AFRICA

## Abstract

**Introduction:**

Clinical governance outlines duties and responsibilities as well as indicators of the actions towards best possible patient outcomes. However, evidence of outcomes on clinical governance interventions is limited in South Africa. This study determined knowledge of clinical staff about the existence of clinical governance protocols/tools that are utilised in selected South African hospitals.

**Methods:**

A cross-sectional study conducted among randomly sampled clinical staff at Nelson Mandela Academic (NMAH), St Elizabeth in the Eastern Cape Province and, Rob Ferreira (RFH) and Themba Hospitals in the Mpumalanga Province of South Africa. A self-administered survey questionnaire was used to collect demographic information and quality improvement protocols/tools in existence at the hospitals. Data were captured in Excel spreadsheet and analysed with STATA. Knowledge was generated based on the staff member’s score for the 12 questions assessed.

**Results:**

A total of 720 participants were recruited of which 377 gave consent to participate. Overall, 8.5% (32/377) of the participants got none or only one correct out of the 12 protocols/tools; and 65.5% (247/377) got between two and five correct. The median knowledge scores were 41.7% (interquartile range (IQR) = 16.7%) in three of the hospitals and 33.3% (IQR = 16.7%) at NMAH (p-value = 0.002). Factors associated with good knowledge included more than five years of experience, being a professional nurse compared to other nurses, not working at NMAH as well as being a medical doctor or pharmacist compared to other staff. Overall, 74.0% (279/377) of the respondents scored below 50%; this was 84.4% (92/109) at NMAH and 66.3% (55/83) at RFH and this difference was statistically significant (p-value = 0.017).

**Conclusion:**

Despite clinical governance implementation, there was low knowledge of clinical governance protocols/tools among clinical staff. Therefore, providing more effective, relevant training workshops with an emphasis on importance of local ownership of the concept of clinical governance, by both management and clinical staff is of great importance.

## Introduction

Clinical governance is a stewardship framework that was popularised by the UK’s National Health Service (NHS) and has been adopted by health institutions globally as an accountability framework for continuously improving the quality of care [1–3]. With its seven pillars; clinical effectiveness, risk management, patient experience and involvement, communication, resource effectiveness, strategic effectiveness, and learning effectiveness, this framework designates roles and responsibilities, and indicators on measures to be taken to ensure optimal patient outcomes [1].

In 2007, the South African National Department of Health (NDoH) submitted an amended policy of quality healthcare addressing challenges faced by hospitals during the provision of quality services [4]. This includes under and overuse of services, avoidable errors, lack of resources, inadequate diagnosis and treatment, inefficient use of resources, an inadequate referral system, disregard for human dignity, drug shortages, records not well kept and poor information, and poor health delivery systems [4]. The policy is in line with the South African National Development Plan (NDP) 2030, to promote change in the quality of healthcare goals and vision as supported worldwide and to reduce disease burden [5]. However, all of the intended policy goals have not been adequately addressed particularly in South Africa’s public health sector [6, 7] and established quality control programs have not been monitored. Overwhelming evidence is reported on a variety of issues that have a negative impact on healthcare quality; such as errors, delays in care delivery, efficacy and costs [7, 8].

To mitigate these setbacks, the NDoH as part of quality assurance, developed the National Core Standards (NCSs) against which service delivery by health establishments can be assessed [9]. The main focus of quality assurance (QA) is to oversee processes, which include adherence to standards and guidelines, or the arrangements and activities meant to safeguard, maintain, and promote quality of care [10]. The standards are not new or additional but combine existing policies and guidelines in a summary form illustrating mandatory organisational requirements and expectations for safe and decent care [9]. The framework is built on values and principles of compassionate care; and provides quality perspectives around health systems, healthcare and health outcomes [11, 12]. The department developed another framework in 2018, the Ideal Hospital Realisation and Maintenance Framework (IHRM-F). This framework is reported to be a critical strategy and intervention to facilitate improved health service delivery and strengthen health system effectiveness by capacitating hospitals to identify and address challenges [13]. The IHRM-F serves as a benchmark mechanism to improve the quality of care by setting and monitoring national norms and standards, improving systems for user feedback, increasing safety in health care, and by improving clinical governance [13]. However, with all these efforts in place, over the past decade, the South African health sector has noted an increase in medico-legal claims in both public and private hospitals [14–17]. Due to patients’ awareness of their constitutional rights, they are now more likely to make medical negligence claims. However, it is not known whether these increasing medico-legal claims in public hospitals can be linked to ineffective protocols, poor implementation of protocols or if frontline health workers are aware of the measures that are in place to improve the quality of care delivered in their hospitals.

Agreement is emerging that a bottom-up approach may be better—where the fundamental motivations of clinical staff are harnessed to drive quality improvement (QI) [18]. This is an appreciation that even though QI and clinical governance are often monitored at the highest level of governance of health facilities, frontline health workers have a significant role to play in ensuring that patient care is of the highest quality [19]. They can play an active role in conducting regular clinical audits, morbidity and mortality, and monitoring of trends [20]. Clinical governance outlines duties and responsibilities as well as indicators of the actions that should be followed to guarantee the best possible patient outcomes. Therefore, the purpose of this study was to ascertain how well-informed clinical staff were about the clinical governance protocols/tools that were utilised in four selected public sector referral hospitals in South Africa.

## Methods

### Study design and setting

A quantitative, cross-sectional study was undertaken from 01 April 2022 to 30 April 2022. The study was conducted in two provinces of South Africa; Eastern Cape (EC) and Mpumalanga (MP). Four hospitals were purposively selected; EC (Nelson Mandela Academic (NMAH) and St Elizabeth Hospitals (SEH)) and MP (Rob Ferreira (RFH) and Themba Hospitals (TH)). Both provinces are regarded as poor provinces [20], with the Eastern Cape ranking as the poorest province at an intensity of 43.3% and Mpumalanga 42.2% [21]. The intensity is an indicator of the relative gap between the median standard of living of the poor population and the poverty line [22]. It makes it possible to see how far the standard of living of the poor population is from the poverty line. The higher this indicator is, the more intense poverty is, in the sense that the standard of living of the poorest is far below the poverty line [22].

### Population and sampling

The target population were all clinical staff; medical doctors, pharmacists, dentists, professional nurses (PNs), enrolled nurses (ENs), enrolled nursing assistants (ENAs), physiotherapists, occupational therapists, speech therapists, audiologists, dieticians, and radiographers from the selected hospitals. The staff were recruited from all hospital departments to determine clinical governance practices implemented in the hospitals. Stratified random sampling was done through a three-stage process. First, a total combined sample size for all four hospitals was calculated using the equation, $n=\frac{p(100-p)z^{2}}{d^{2}}$ for a one-sided 95% confidence interval and a 5% significance level (z = 1.96). Because the proportion (p) of clinical governance information available was not known, the (p) was set at 50% and the desired precision (d) was set at 4% to yield a minimum sample size of 600 participants. To factor for data entry errors a further 20% (120) was added to yield a desired sample size of 720 participants for all four study sites.

Second, the clinical staff from the four hospitals as of August 2022 were added together to provide the total population size (N = 2492), wherein RFH = 712; TH = 480; NMAH = 900; and SEH = 400. The weighted hospital sample size was calculated based on the equation, *sample _hy = hy N x* 720 where y is the value 1 to 4 depending on the hospital being calculated. Third, clinical staff were allocated into strata based on their profession, this allowed for a calculation of the strata specific sample per hospital. The calculation was similar to that of calculating the hospital specific sample as above. The hospitals’ human resources department assisted with the listing of staff, to ensure compliance with the country’s privacy laws. Staff were randomly selected per strata based on their listings and approached for participation in the study.

### Data collection

A validated self-administered survey questionnaire consisting of questions on demographic characteristics and clinical governance activities that they knew to take place or be present in their hospitals. S1 Appendix summarises the quality improvement activities assessed. S2 Appendix presents the actual present or absent clinical governance policies or practices in each of the four hospitals.

### Data analysis

Data were captured and coded in Microsoft Excel and analysed with Stata version 18.0. Numerical data were explored for normality using the Shapiro Wilk test. The median and interquartile range (75^th^ percentile– 25^th^ percentile) were used to summarise participants’ age in years and years of experience. Categorical variables were summarised using frequencies, percentages and graphs. The Kruskal-Wallis test was used to compare the equality of median ages and years of experience of participants by hospital. Categories were compared using the Chi-squared test, but when the expected frequencies were <5 the Fisher’s exact test was used (Tables 1 and 2). Individual health workers’ responses were compared to the evidence supplied by that hospital on the presence or absence of the policies or practices and scored. Knowledge was generated based on the score being either below 50% or greater than or equal to 50% for the 12 questions assessed. Since scores were not normally distributed, the median and interquartile range (IQR) are used to summarise the knowledge scores between the facilities and compared using the Kruskal Wallis test. Binomial logistic regression models were used to determine associations of a positive response. The prevalence ratio (PR) is the measure of association used. To show the precision of estimates, 95% confidence intervals (95%CI) are reported, and the p-value used for statistical significance is, p-value≤0.05.

**Table 1:** Participants’ basic characteristics.

Characteristics		Overall		TH		NMAH		RFH		SEH		p-value
Hospital, n (%)		377	(100.0)	61	(16.2)	109	(28.9)	83	(22.0)	124	(32.9)	-
Sex, n (%)												
	Female	310	(82.2)	46	(75.4)	90	(82.6)	5	(6.0)	96	(77.4)	0.008
	Male	67	(17.8)	15	(24.6)	19	(17.4)	78	(94.0)	28	(22.6)	
Profession, n (%)												
	Doctor	51	(13.5)	8	(13.1)	20	(18.3)	6	(7.2)	17	(13.7)	<0.001
	Pharmacist	14	(3.7)	8	(13.1)	5	(4.6)	0	(0.0)	1	(0.8)	
	PN	184	(48.8)	28	(45.9)	54	(49.5)	54	(65.1)	48	(38.7)	
	EN	57	(15.1)	7	(11.5)	10	(9.2)	18	(21.7)	22	(17.7)	
	ENA	53	(14.1)	1	(1.6)	18	(16.5)	4	(4.8)	30	(24.2)	
	Allied*	18	(4.8)	9	(14.8)	2	(1.8)	1	(1.2)	6	(4.8)	
Age^α^, years; med (IQR)		40.2	(16.5)	34.0	(18.3)	43.8	(19.6)	39.7	(14.3)	38.7	(15.2)	0.004^^^
Age^α^, years; n (%)												
	<30	38	(15.4)	9	(37.5)	4	(5.7)	5	(17.9)	20	(16.1)	0.003
	30–39	78	(31.7)	6	(25.0)	20	(28.6)	9	(32.1)	43	(34.7)	
	40–49	75	(30.5)	4	(16.7)	20	(28.6)	9	(32.1)	42	(33.9)	
	50–64	55	(22.4)	5	(20.8)	26	(37.1)	5	(17.9)	19	(15.3)	
Experience^ß^, years; med (IQR)		8	(10.0)	8	(9.0)	10	(10.0)	8	(14.0)	7.5	(9.0)	0.133
Experience^ß^, years; n (%)												
	≤1	36	(10.3)	11	(19.6)	6	(5.9)	6	(9.1)	13	(10.5)	0.063
	1.1–5	89	(25.6)	8	(14.3)	26	(25.5)	17	(25.7)	38	(30.6)	
	6–10	92	(26.4)	17	(30.4)	26	(25.5)	18	(27.3)	31	(25.0)	
	11–20	95	(27.3)	13	(23.2)	30	(29.4)	15	(22.7)	37	(29.8)	
	21–37	36	(10.3)	7	(12.5)	14	(13.7)	10	(15.2)	5	(4.0)	

TH = Themba Hospital; NMAH = Nelson Mandela Academic Hospital; RFH = Rob Ferreira Hospital; SEH = St Elizabeth Hospital; PN = Professional Nurse; EN = Enrolled Nurse; ENA = Enrolled Nurse Assistant

*(Physiotherapist, n = 3; Occupational Therapist, n = 5; Speech Therapist, n = 1; Audiologist, n = 1; Dietician, n = 5)

^α^n = 246

^ß^n = 348

^^^Kruskal Wallis test was used.

**Table 2:** Participants’ responses on protocols and practices in their hospitals.

Characteristics		Overall		TH		NMAH		RFH		SEH		p-value
Complaints^α^; n (%)												
	Yes	277	(75.9)	47	(79.7)	63	(63.0)	58	(69.9)	109	(88.6)	0.000
	No	20	(5.5)	5	(8.5)	11	(11.0)	2	(2.4)	5	(8.5)	
	Not sure	68	(18.6)	7	(11.9)	26	(26.0)	23	(27.7)	7	(11.9)	
Adverse Events^ß^; n (%)												
	Yes	279	(75.4)	47	(79.7)	64	(61.5)	63	(75.9)	105	(84.7)	0.007
	No	23	(6.2)	2	(3.4)	10	(9.6)	6	(7.2)	5	(4.0)	
	Not sure	68	(18.4)	10	(17.0)	30	(28.8)	14	(16.9)	14	(11.3)	
Morbidity and Mortality*^^^; n (%)												
	Yes	236	(65.7)	42	(71.2)	55	(53.9)	57	(72.2)	82	(68.9)	0.154
	No	28	(7.8)	5	(8.5)	10	(9.8)	5	(6.3)	8	(6.7)	
	Not sure	95	(26.5)	12	(20.3)	37	(36.3)	17	(21.5)	29	(24.5)	
Regular reviews^α^; n (%)												
	Yes	244	(66.8)	32	(54.2)	65	(63.7)	57	(68.7)	90	(74.4)	0.141
	No	29	(79.4)	7	(11.9)	9	(8.8)	4	(4.8)	9	(7.4)	
	Not sure	92	(25.2)	20	(33.9)	28	(27.5)	22	(26.5)	22	(18.2)	
Centralised^α^; n (%)												
	Yes	222	(60.8)	44	(74.6)	59	(57.8)	42	(51.9)	77	(62.6)	0.201
	No	35	(9.6)	3	(5.1)	12	(11.7)	10	(12.3)	10	(8.1)	
	Not sure	108	(29.6)	12	(20.3)	31	(30.4)	29	(35.8)	36	(29.3)	
Development^♣^; n (%)												
	Yes	235	(64.7)	41	(71.9)	64	(63.4)	48	(57.8)	82	(67.2)	0.223**
	No	52	(14.3)	8	(14.0)	10	(9.9)	14	(16.9)	20	(16.4)	
	Not sure	76	(20.9)	8	(14.0)	27	(26.7)	21	(25.3)	20	(16.4)	
IPC^Ҧ^; n (%)												
	Yes	292	(79.6)	46	(78.0)	84	(80.8)	61	(73.5)	101	(83.5)	0.167
	No	22	(6.0)	7	(11.9)	3	(2.9)	7	(8.4)	5	(4.1)	
	Not sure	53	(14.4)	6	(10.2)	17	(16.3)	15	(18.1)	15	(12.4)	
Clinical Protocols^ʊ^; n (%)												
	Yes	250	(68.3)	42	(72.4)	66	(64.1)	44	(53.0)	98	(80.3)	0.001
	No	20	(5.5)	1	(1.7)	4	(3.9)	8	(9.6)	7	(5.7)	
	Not sure	96	(26.2)	15	(25.9)	33	(32.0)	31	(37.3)	17	(13.9)	
Care coordination^ɸ^; n (%)												
	Yes	201	(56.5)	29	(51.8)	54	(52.9)	43	(52.4)	75	(64.7)	0.022
	No	33	(9.3)	1	(1.8)	14	(13.7)	6	(7.3)	12	(10.3)	
	Not sure	122	(34.3)	26	(46.4)	34	(33.3)	33	(40.2)	29	(25.)	
Safety^##^; n (%)												
	Yes	262	(74.0)	45	(78.9)	63	(64.3)	60	(74.1)	94	(79.7)	0.030
	No	32	(9.0)	4	(7.0)	8	(8.2)	12	(14.8)	8	(6.8)	
	Not sure	60	(16.9)	8	(14.0)	27	(27.6)	9	(11.1)	16	(13.6)	
Information system^ж^; n (%)												
	Yes	152	(43.6)	21	(38.2)	40	(42.6)	34	(42.0)	57	(47.9)	0.742**
	No	90	(25.8)	17	(30.9)	27	(28.7)	22	(27.2)	24	(20.2)	
	Not sure	107	(30.7)	17	(30.9)	27	(28.7)	25	(30.9)	38	(31.9)	
Health education ^ß^; n (%)												
N = 353	Yes	239	(67.7)	38	(69.1)	58	(59.8)	50	(62.5)	93	(76.9)	0.181
	No	55	(15.6)	9	(16.4)	19	(19.6)	15	(18.8)	12	(9.9)	
	Not sure	59	(16.7)	8	(14.5)	20	(20.6)	15	(18.8)	16	(13.2)	

IPC = Infection Prevention and Control; TH = Themba Hospital; NMAH = Nelson Mandela Academic Hospital; RFH = Rob Ferreira Hospital; SEH = St Elizabeth Hospital

^α^n = 365

^ß^n = 370; *

^^^n = 359

^♣^n = 363; ^Ҧ^n = 367

^ʊ^n = 366

^ɸ^n = 356

^##^n = 354

^ж^n = 349

^ß^n = 353

**Chi-squared test was used

### Ethical consideration

Ethical clearance was attained from the Witwatersrand University, Human Research Ethics Committee (HREC) [M210939], and Walter Sisulu University [040/2021]. Permission for data collection was granted by the Eastern Cape and Mpumalanga Provincial Health Research Committees and the hospital management of the four hospitals. All participants provided written, informed consent. Data were treated as confidential, and participants’ names were anonymised.

## Results

Three hundred and seventy-seven (377) participants gave consent to participate in the study. Of these, 32.9% (n = 124) worked at SEH and 16.2% (n = 61) worked at Themba hospital. More than three quarters of the participants were female. Overall, professional nurses comprised 48.8% of the participants, and the median age was 40.2 years, with the oldest participant being 64 years old. Except for the years of experience, all other comparisons were statistically different between the four health facilities (p-value<0.008). Demographic characteristics are summarised in Table 1.

### Confirmation of presence of protocols/tools and knowledge scores

Participants were asked about the existence of selected protocols, guidelines or practices within their hospitals (Table 2). Whilst 75.9% (277/365) of all respondents were affirmative that their hospitals had complaints management protocols, only 63.0% (63/100) of NMAH’s respondents and 88.6% (109/121) of SEH’s respondents were respectively positive about the presence of these protocols in their hospital. There was a statistically significant difference in reporting the presence of complaints management protocols across the different hospitals (p-value<0.0001). Likewise, 75.4% of all respondents were positive about the presence of adverse events protocols in their hospitals, 61.5% (64/104) and 84.7% (105/124) of NMAH’s and SEH’s respondents were respectively positive about the presence of this protocol (p-value = 0.007).

All individual staff responses were compared with the actual hospital’s reports; obtained from the CEO and Quality Assurance offices, on the presence or absence of the 12 protocols assessed (Table 3). Overall, 8.5% (32/377) of the participants got none or only one correct out of the twelve protocols; and 65.5% (247/377) got between two and five out of twelve correct. The differences between the hospitals were statistically significant (p-value = 0.002) with 31.1% (19/61) and 14.7% (16/109) of Themba hospital and NMAH respondents respectively getting between six and eight correct. Also noted in Table 3 is that 74.0% (279/377) of overall respondents scored below 50%; this was 84.4% (92/109) at NMAH and 66.3% (55/83) at RFH and this difference was statistically significant (p-value = 0.017).

**Table 3:** Staff knowledge of policies or practices that were available within their hospitals.

Characteristics		Overall		TH		NMAH		RFH		SEH		p-value
Score, perc; med (IQR)		41.7	16.7	41.7	16.7	33.3	16.7	41.7	16.7	41.7	16.7	0.002^#^
Score, n (%)												
	0–1	32	8.5	6	9.8	15	13.8	10	12.0	1	0.8	0.002
	2–5	247	65.5	35	57.4	77	70.6	45	54.2	90	72.5	
	6–8	90	23.9	19	31.1	16	14.7	26	31.3	29	23.4	
	9–11	8	2.1	1	1.6	1	0.9	2	2.4	4	3.2	
Score categories; n (%)												
	<50%	279	(74.0)	41	(67.2)	92	(84.4)	55	(66.3)	91	(73.4)	0.017*
	> = 50%	98	(26.0)	20	(32.8)	17	(15.6)	28	(33.7)	33	(26.6)	

TH = Themba Hospital; NMAH = Nelson Mandela Academic Hospital; RFH = Rob Ferreira Hospital; SEH = St Elizabeth Hospital; *Chi-squared test used

^#^ Kruskal Wallis test used.

**Fig 1:**
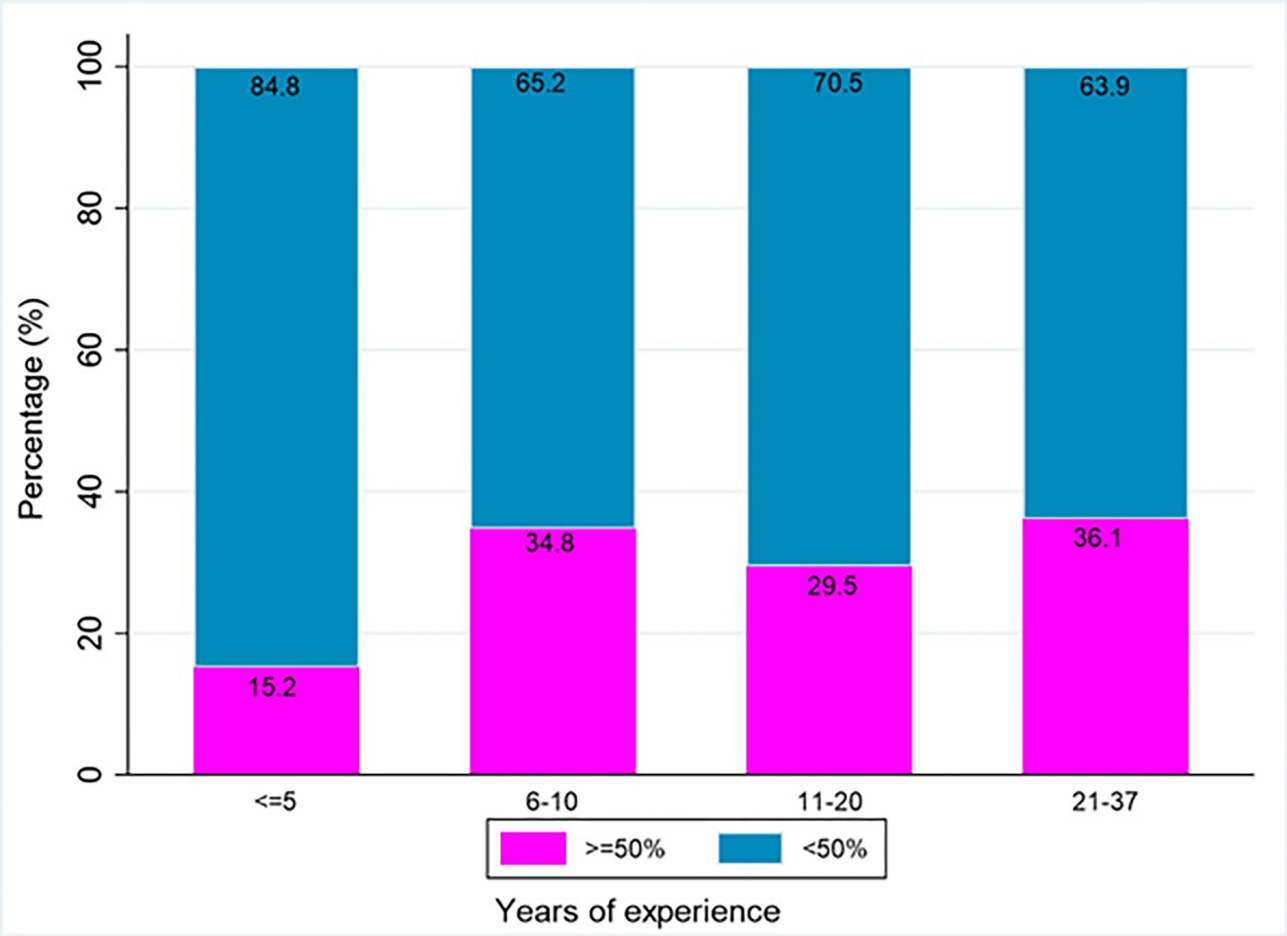
Staff knowledge score percentages by years of experience.

Further noted in Table 3 and Fig 1, is that the median knowledge scores were 41.7% (25^th^ percentile = 33.3% and 75^th^ percentile = 50.0%) in three of the hospitals, and 33.3% (25^th^ percentile = 25.0% and 75^th^ percentile = 41.7%) at NMAH. These differences were statistically significant (p-value = 0.002).

### Association between demographic factors and knowledge

**Fig 2:**
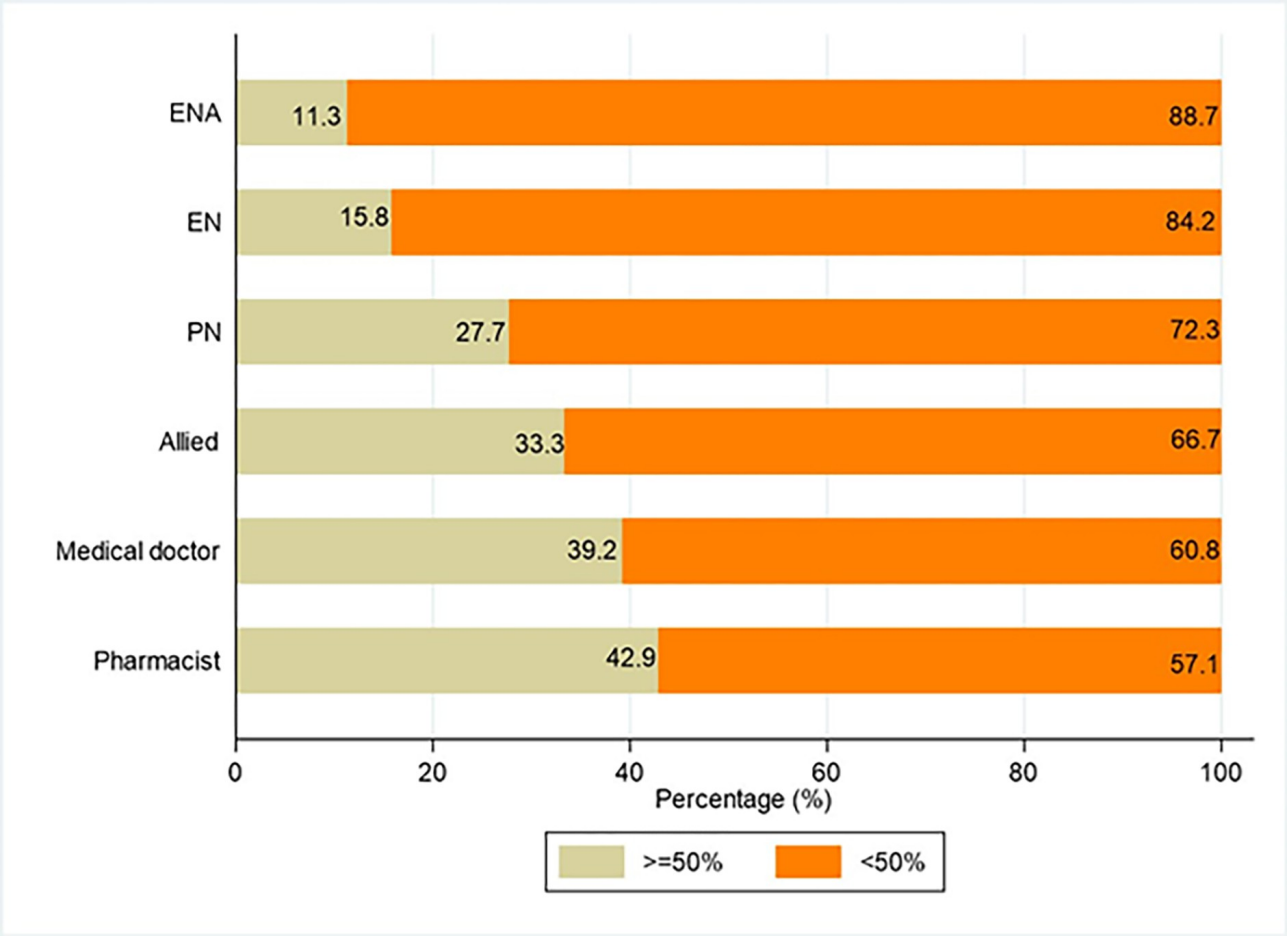
Percentage of staff who were affirmative about all protocols/practices in their hospitals by profession. TH = Themba Hospital; NMAH = Nelson Mandela Academic Hospital; RFH = Rob Ferreira Hospital; SEH = St Elizabeth Hospital.

Fig 2 shows that only 15.2% (19/125) of those who had not reached five years of experience in that health facility had a knowledge of at least 50% of the available policies or practices, compared to 36.1% for those who had worked for between 21 and 37 years.

**Fig 3:**
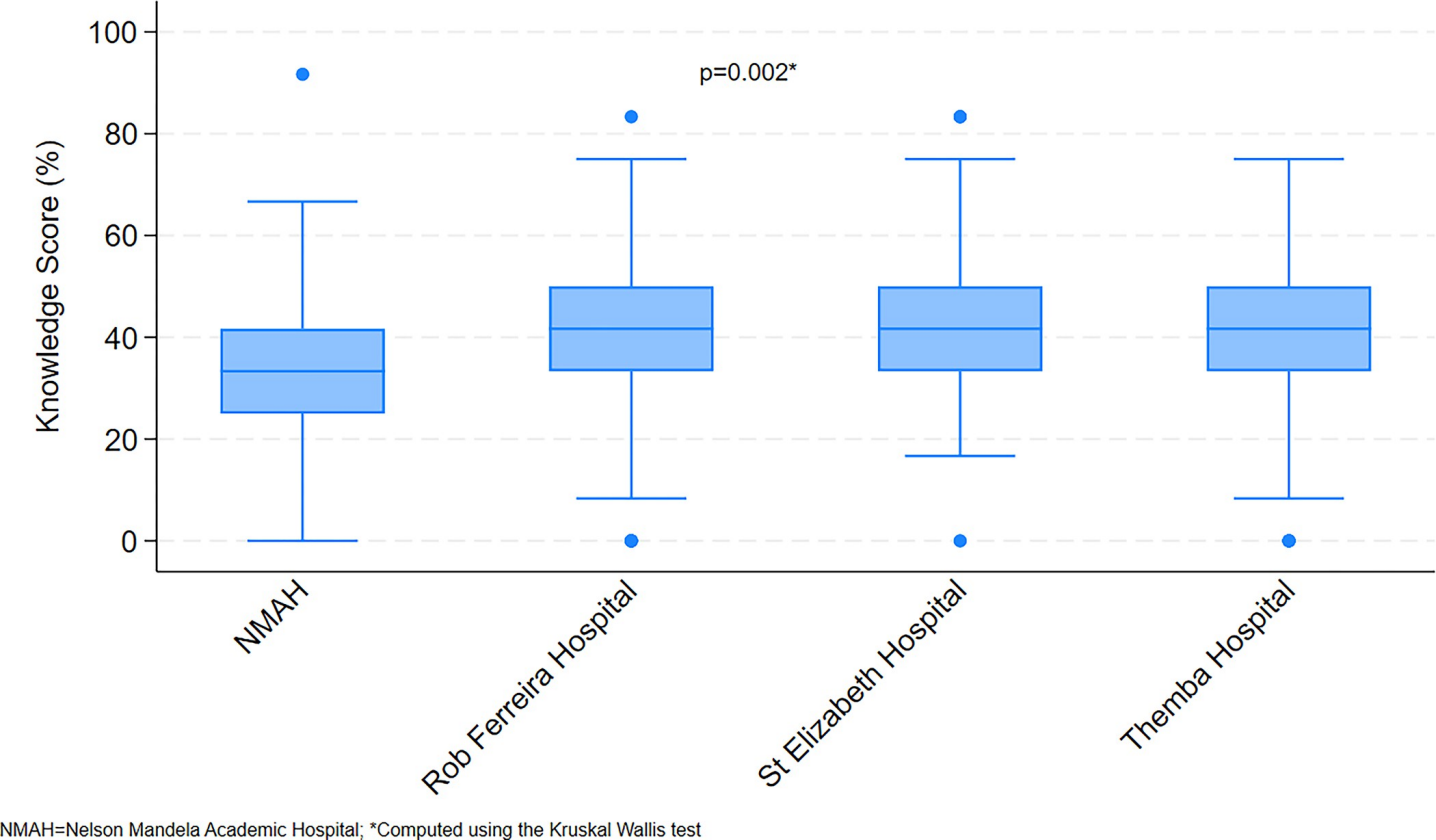
Percentage of staff who were knowledgeable about all protocols/practices in their hospitals by hospital.

Whilst only 27.7% (51/184) of PNs knew of the existence of at least 50% of the present policies or practices, 42.9% (6/14) and 39.2% (20/51) of pharmacists and doctors verified at least half of the policies or practices (Fig 3).

Table 4 shows that individuals who had more than five years of experience were at least 20% more likely to have gotten 50% or more of the policies or practices correct compared to those with five years or less and these were statistically significant (p<0.05). Whilst PNs were 20% more likely to know at least 50% of the existing policies or practices than other nursing categories (PR = 1.2; 95%CI: 1.1–1.4; p-value = 0.002), medical doctors were 30% more likely to have fared better than the aggregated nursing categories and this was statistically significant (PR = 1.3; 95%CI: 1.0–1.6; p-value = 0.037).

**Table 4:** Bivariable associations of knowledge on protocols/practices in their hospitals.

Characteristics	n/N	Prevalence Ratio	95%CI	p-value
**Sex**				
Male	22/67	1	ref	ref
Female	76/310	0.8	(0.7–1.1)	0.201
Age, years				
<40	29/116	1	ref	ref
40–64	28/130	1.0	(0.8–1.1)	0.523
Level of experience, years				
≤5	19/125	1	ref	ref
6–10	32/92	1.3	(1.1–1.5)	0.002
11–20	28/95	1.2	(1.0–1.4)	0.016
21–37	13/36	1.3	(1.0–1.7)	0.031
Hospital				
NMAH	17/109	1	ref	ref
RFH	28/83	1.3	(1.1–1.5)	0.006
SEH	33/124	1.2	(1.0–1.3)	0.040
TH	20/61	1.3	(1.0–1.5)	0.021
Nurse				
ENA	6/53	1	ref	ref
EN	9/57	1.1	(0.9–1.2)	0.493
PN	51/184	1.2	(1.1–1.4)	0.002
Profession				
Nurse	66/294	1	ref	ref
Medical doctor	20/51	1.3	(1.0–1.6)	0.037
Pharmacist	6/14	1.4	(0.9–2.1)	0.191
Allied	6/18	1.2	(0.8–1.6)	0.373

95%CI = 95% Confidence interval

## Discussion

This study determined the knowledge of clinical staff on clinical governance protocols/tools that are available and/or utilised in their hospitals. The research found a majority of participants had sub-optimal knowledge of the protocols or tools that existed in their hospitals. Complaints management and adverse events protocols were two, out of twelve, most known and positively affirmed as available and in use at all facilities. Factors associated with good knowledge of the protocols/tools included more than five years of experience, being a professional nurse compared to other nurses, not working at NMAH as well as being a medical doctor or pharmacist compared to other staff.

In South Africa, the concept of clinical governance has been introduced into different policy frameworks [23, 24]. This study and literature reveal that the drive to improve the quality of healthcare in South Africa has not been lacking in interventions and powerful ideas [25]. However, there is limited literature about evaluating clinical governance implementation and its impact in South Africa. This cross-sectional study is one of the first to determine clinical staff knowledge of clinical governance protocols/tools in selected South African public hospitals. It is reported that creating a sense of ownership, use of educative methods, information sharing, use of procedures and clinical guidelines are some of the effective activities in better implementation of clinical governance [26]. However, the results of this study demonstrate that clinical governance is implemented in these hospitals, but with a concerningly low level of staff knowledge about the concepts of clinical governance. This study therefore validates the importance of ensuring that protocols and guidelines are not only available as a tick box but also ensuring that the mission and vision of hospitals operationalise these protocols and guidelines. In this way, all frontline health workers will be aware of these protocols and guidelines and as such they will have the desired impact.

The results of this study are similarly reported for public hospitals in Tehran [26]. The two mostly known and affirmed protocols; complaints management and adverse events, could be due to more emphasis as these are key priority areas [9, 13]. Which is an emphasis of the continuous intervention for quality improvement and responsibility in all health establishments [27]. These results highlight limited or lack of information sharing on the concept of clinical governance as well as possible non-achievement of desired outcomes to improve the quality of care in their facilities.

Literature [28] has long established that, in order to improve the quality of healthcare, knowledge must be applied. Quality improvement seeks to standardise processes and structures to reduce variation, achieve predictable results, and improve outcomes for patients, health systems, and organisations [29]. Although much has been done over the years to restructure the system and improve the quality of care in health systems, literature reveals that millions of people in South Africa still suffer preventable harm every day [8]. Medical litigation has dramatically increased both in frequency and in the size of the damages [30] reflecting the cost of poor knowledge and application of important protocols or guidelines by frontline workers.

Participants from NMAH had the least knowledge of clinical governance protocols/tools compared to other facilities studied. This continues to cause long delays in the achievement of quality healthcare delivery [31]. Considering that NMAH is an academic quaternary hospital, a better knowledge was expected. The least knowledge of protocols in this academic facility may point to a deeper problem with regards to leadership as previously observed in another publication [32]. Experience and the type of profession were associated with a higher knowledge of the protocols/tools. Despite professional nurses not being fully knowledgeable of existing clinical governance protocols/tools in their facilities, they were more knowledgeable compared to other nursing categories. This could be through repeated exposure and building of a culture that incorporates these protocols into everyday practice, they have become more knowledgeable while other categories are still under supervision. One of the firm foundations for the structure of clinical governance is education and training to raise the awareness of the nature of clinical governance [33, 34]. Therefore, lack of introductory training on clinical governance could have been a significant factor in junior staff having less knowledge about the protocols. Allied (physiotherapist, dieticians etc.) workers were either unsure or didn’t think the protocols and practices were present, this could have been due to the fact that they do not interact with some. Doctors and Pharmacists had better knowledge than other professions. Previous studies have shown that separate meetings and reporting forums for clinical and nursing managers, under the leadership of the Chief Executive Officer (CEO), could be the sole reason for differences between frontline health workers on knowledge of clinical governance tools in their hospital [35].

The design of this study could not allow for a conclusion on the causality of clinical governance protocol/tools knowledge or lack thereof. The use of the quantitative method limited this study in that it typically focused on objective data and did not capture the subjective experiences (deeper insights) of participants about clinical governance implementation at their hospitals. Regardless of the limitations, this study established preliminary evidence for planning of an intervention. Clinical staff often have a first-hand and positive experience of improving quality of care [36]. Therefore, clinical staff in these hospitals need to take the initiative and responsibility in the development and/or review of clinical governance protocols/tools in these hospitals. Managers of the facilities must prioritise clinical governance information sharing with clinical staff. Workshops are recommended to better understand the clinical governance framework, clarify the roles and responsibilities for all players and establish structures of quality improvement in the hospitals. Training workshops must put emphasis on importance of local ownership of the concept of clinical governance, by both management and clinical staff [37]. Communication and clinical governance information sharing amongst clinicians, line managers and policymakers should be at the top of priorities. Junior staff must be introduced to clinical governance in curriculum and during employment induction. The latter must be a practice for all new clinical employees.

## Conclusion

The concepts and goals of clinical governance have not been effectively conveyed to frontline health workers in these hospitals. Despite clinical governance implementation, there has been low clinical governance protocols or tools knowledge among the staff. Therefore, providing more effective relevant training workshops, with emphasis on the importance of local ownership of the concept of clinical governance by both management and clinical staff will likely improve knowledge on clinical governance and the quality of care rendered. Clinical Governance must be implemented through comprehensive management support and participation of all staff and health professionals. For junior staff with less than five years of experience it is recommended that they must be introduced to clinical governance tools in their curricula and during induction. Any plans that come out of the intervention must be designed to be sustainable and implementable within current resources and other facilities. Focus areas are to prioritise communication between management and clinical staff and set up measures for the impact of the intervention.

## Supporting information

S1 Appendix Clinical Governance Implementation Status [CGISS] questionnaire for clinical staff. (PDF)

S2 Appendix Confirmation of presence of quality improvement activities by management in each hospital. (PDF)

S3 Appendix Information sheet for clinical staff. (PDF)

S1 Data Manuscript data file. (DTA)
