# Supplementary material for: Clinical staff reported knowledge on the existence of clinical governance protocols or tools utilised in selected South African hospitals
Source: PLoS One. 2024 Nov 21;19(11):e0312340. doi: 10.1371/journal.pone.0312340 (PMC11581235; doi:10.1371/journal.pone.0312340)
Supplement: S1 Appendix — (PDF) [file pone.0312340.s001.pdf]

S1 Appendix A

Clinical Governance Implementation Status [CGISS] questionnaire for clinical staff

Subject Number: ___________________________

Date of Administration: ________________________________

Tick the correct response.

DEMOGRAPHIC PROFILE

Nationality	1 South Africa-born<br>2 Non-South Africa-born
Sex	1 Male<br>2 Female
Date of birth dd/mm/yyyy	………/……./……………….
Years of service	_________________
Name of hospital	1 Nelson Mandela Academic Hospital<br>2 Rob Ferreira Hospital<br>3 St Elizabeth Hospital<br>4 Themba Hospital
Organisational status	1 Medical doctor<br>2 Pharmacist<br>3 Dentist<br>4 Professional nurse<br>5 Enrolled nurse6 Enrolled nursing assistant<br>7 Physiotherapist<br>8 Occupational therapist<br>9 Speech Therapist<br>10 Audiologist<br>11 Dietician<br>12 Radiographer

Confirmation of presence of quality improvement protocols/tools/activities.
Please check one column for each statement using the following codes: 1= yes, 2= no

**Quality Improvement Activity**	**Yes (1)**	**No (2)**	**Comment and supply evidence** (Protocol from Province and National and SOP)
1. A Complaints Reporting System			
2. An Adverse Events Reporting System (with PSI)			
3. Scheduled Mortality & Morbidity Review Meetings			
4. Regular review of a sample of patient records			
5. Centralised File of all Hospital Policies and Procedures			
6. In-house continuing Clinical Development & Education Programme			
7. In-house Infection Prevention and Control Programme			
8. Clinical protocols for common conditions across relevant clinical areas			
9. Systems of care coordination across all clinical areas			
10. In-house Occupational Health & Safety Programme			
11. Computerised health management information system			
12. Health Education Programme across all clinical areas			
13. Clinical Governance protocol			
