# Supplementary material for: Clinical staff reported knowledge on the existence of clinical governance protocols or tools utilised in selected South African hospitals
Source: PLoS One. 2024 Nov 21;19(11):e0312340. doi: 10.1371/journal.pone.0312340 (PMC11581235; doi:10.1371/journal.pone.0312340)
Supplement: S2 Appendix — (PDF) [file pone.0312340.s002.pdf]

S2 Appendix B

Confirmation of presence of quality improvement activities by management in each hospital

Quality Improvement Activity	NMAH	FRH	TH	SEH
1. A Complaints Reporting System	Yes	Yes	Yes	Yes
2. An Adverse Events Reporting System	Yes	Yes	Yes	Yes
3. Mortality & Morbidity Review Meetings	No	No	No	No
4. Regular review of a sample of patient records	No	No	No	No
5. Centralised File of all Hospital Policies	No	No	No	No
6. Education Protocol	No	No	No	No
7. IPC Protocol	Yes	Yes	Yes	Yes
8. Clinical Guidelines Protocol	No	No	No	No
9. Systems of care coordination	No	No	No	No
10. Health & Safety Programme	Yes	Yes	Yes	Yes
11. Computerised health information system	No	No	No	No
12. Health Education Programme across areas	No	No	No	No
13. Clinical Governance protocol	No	No	No	No

Document completed by (position): ____

Signature____

Date of completion____
