# Supplementary material for: Clinical staff reported knowledge on the existence of clinical governance protocols or tools utilised in selected South African hospitals
Source: PLoS One. 2024 Nov 21;19(11):e0312340. doi: 10.1371/journal.pone.0312340 (PMC11581235; doi:10.1371/journal.pone.0312340)
Supplement: S3 Appendix — (PDF) [file pone.0312340.s003.pdf]

S3 Appendix C:

Information sheet for Clinical Staff

Title: Exploring the feasibility of improving the performance of public hospitals through a focused implementation clinical governance protocol in South Africa's Eastern Cape and Mpumalanga provinces

Dear Sir or Madam

Purpose of the study: This study aims to assess the effectiveness of clinical governance interventions in four public hospitals in South Africa's Eastern Cape and Mpumalanga provinces. We want to understand your perspective in relation to the delivery of healthcare in the selected hospitals from both the Eastern Cape and Mpumalanga provinces.

Procedure to be followed: Data will be collected in the form of completion of a questionnaire. Data will be conducted in Eastern Cape province (Nelson Mandela Academic hospital & St Elizabeth hospital) and Mpumalanga province (Rob Ferreira hospital & Themba hospital). The data collection will be conducted between January 2022 to July 2022. COVID-19 related regulations will be followed during the collection of data.

Benefits: There are no direct benefits to you. You will not receive money or any other incentives to take part in this study. Even though study findings will not be of direct benefit to you, it is hoped that by participating in this study, you will assist in identifying factors that act as barriers/enablers to improving performance of hospitals.

Risks: The risks in this study are minimal (i.e., no greater than those ordinarily encountered in daily life). There are no foreseeable discomforts or dangers to you. There will be no cost to you for participating in this study.

Duration of participation: Participation in the study may not exceed 30 minutes.

Approval of the study: Ethical clearance was obtained from the Research Ethics Committees of the University of the Witwatersrand and Walter Sisulu University. Permission to access the

health facilities were obtained from provincial research authorities of both the Eastern Cape and Mpumalanga, and from the Chief Executive Officer of the selected hospitals.

Anonymity and confidentiality: All records will be kept confidential and will be available only to professional researchers and staff. If the results of this study are published, the data will be presented in group form and individual or personal identifiers of participants will not be used.

Voluntary participation: Your participation is voluntary, and you are free to withdraw at any time if you wish to do so.

Termination of participation: If at any point during the study you wish to terminate the session, we will do so. If any of the questions from the study causes you distress, the study will be terminated immediately, and counselling will be provided at no cost to you by a registered health professional in the hospital.

Outputs: If you would like to be informed of the final research findings, please contact the principal investigator (contact details at the end of this information sheet). Findings will be disseminated widely to all stakeholders, annual partner meetings, peer review journals, national and international conferences.

This study has been approved by the Human Research Ethics Committee (Medical) of the University of the Witwatersrand, Johannesburg ("Committee"). A principal function of this Committee is to safeguard the rights and dignity of all human subjects who agree to participate in a research project and the integrity of the research.
If you have any concern over the way the study is being conducted, please contact the Chairperson of this Committee who is Dr Clement Penny, who may be contacted on telephone number 011 717 2301, or by e-mail on Clement.Penny@wits.ac.za. The telephone numbers for the Committee secretariat are 011 717 2700/1234 and the e-mail addresses are Zanele.Ndlovu@wits.ac.za and Rhulani.Mukansi@wits.ac.za.

Principal investigator: Dr Wezile Chitha, Principal Investigator, telephone number 011 084 4901, or by e-mail at wchitha@witshealth.co.za.

Thank you for reading this Study Information Sheet.
